# The Intergovernmental Panel on Climate Change overestimates annual nitrogen excretion from pigs in Korea

**DOI:** 10.5713/ab.25.0586

**Published:** 2025-10-22

**Authors:** Donghyeon Lee, Hansol Kim, Jongkeon Kim, Beob Gyun Kim

**Affiliations:** 1 Department of Animal Science, Konkuk University, Seoul, Korea; 2 Department of Animal and Food Sciences, University of Kentucky, Lexington, KY, USA; 3 Swine Innovation Center, Sunjin Company Limited, Seoul, Korea

**Keywords:** Intergovernmental Panel on Climate Change, Nitrogen Excretion, Representative Body Weight, Swine

## Abstract

**Objective:**

This study aimed to validate the accuracy of representative body weight (BW) and annual nitrogen (N) excretion (Nex) of pigs suggested by the Intergovernmental Panel on Climate Change (IPCC) and to estimate the annual Nex from pigs in Korea.

**Methods:**

Seven crossbred barrows (initial BW = 56.6±2.3 kg) were used to determine apparent total tract digestibility (ATTD) of dry matter and N, and the fresh-to-dry fecal weight ratio. Daily BW, feed intake, and age data were collected from the National Research Council and the Korean Feeding Standard for Swine. Models for daily BW, feed intake, and fresh fecal excretion of pigs were developed to calculate the representative BW. Daily fecal Nex from a market pig was calculated using ATTD of N and daily N intake, and daily urinary Nex was estimated. Total Nex was calculated as the sum of fecal and urinary Nex.

**Results:**

The ATTD of dry matter and N was 88.4% and 88.8%, respectively, and the fresh-to-dry fecal weight ratio was 3.55. Based on the average daily feed intake of 1,602 g/day and fresh fecal excretion of 587 g/day, the representative BW for market pigs was determined to be 35.3 kg at 85 days of age. The daily fecal, urinary, and total Nex at the representative BW were 5.6, 15.3, and 20.9 g/day, respectively. The annual total Nex for a market pig was 7.6 kg/year. The annual Nex for a reproductive sow was 14.5 kg/year. Assuming a population of 91% market pigs and 9% breeding sows, the annual total Nex for all pigs in the Korean swine industry was 8.23 kg/year which is less than 18.0 kg/year suggested by the IPCC (p<0.001).

**Conclusion:**

The IPCC overestimated the representative BW of pig and Nex from pigs in Korea. The annual Nex from pigs is 8.23 kg/year in Korea.

## INTRODUCTION

Nitrogen (N) is a component of proteins and amino acids that are essential for maintenance and optimal growth of pigs [1]. Dietary N is used for physiological functions and protein deposition in pigs, but undigested and unutilized N is excreted in feces and urine, respectively. Excreted N is converted into nitrous oxide (N_2_O) during pig manure storage and composting [2,3]. The N_2_O is one of the major greenhouse gases in the livestock industry, raising concerns about environmental pollution and climate change [4,5].

To efficiently manage N_2_O emissions from swine production, an accurate determination of annual N excretion (Nex) from pigs is essential. The Greenhouse Gas Inventory and Research Center of Korea [6] adopted the annual Nex of 20 kg/year from pigs that was suggested by the Intergovernmental Panel on Climate Change (IPCC) for Western Europe [7] in 1996. As the annual Nex from pigs suggested by the IPCC was estimated in the 1990s, this value may not reflect the recent rearing conditions of pigs. In 2019, the IPCC [8] proposed a method for estimating annual Nex using the daily Nex rate (kg/1,000 kg of animal mass) and the representative body weight (BW) of pigs. The representative BW refers to the average BW of pigs over a year. The IPCC [8] calculated annual Nex by dividing the daily Nex rate by the representative BW and then multiplying by 365 days. However, the daily Nex rate and the representative BW of pigs provided by the IPCC [8] have not been validated for pigs raised in Korea. Therefore, the objectives of the present study were to validate the representative BW of market pigs, the daily Nex rate, and the annual Nex suggested by the IPCC and to estimate country-specific values for the Korean swine industry.

## MATERIALS AND METHODS

### Animals, diets, and sample collection

A total of 7 barrows ([Landrace×Yorkshire]×Duroc) with initial BW of 56.6 kg (standard deviation = 2.3) were used to determine apparent total tract digestibility (ATTD) of dry matter (DM) and N, and fresh-to-dry fecal ratio. The animals were individually housed in metabolism crates equipped with a feeder. All pigs were fed a commercial diet (Nonghyup Feed; Table 1) in a daily amount of 3.0 times the estimated energy requirement for maintenance (i.e., 197 kcal of metabolizable energy per kg BW^0.60^; [1]). The daily feed allowance per pig was divided into two equal meals and fed at 0800 and 1700 h. Water was freely available.

The experimental period consisted of 5 days of adaptation to the commercial diet and 5 days of fecal collection. Chromic oxide was added at 5 g per kg of morning meals on days 6 and 11 of the period as an indigestible marker for the initiation and termination of fecal collection, respectively [9]. The collection was initiated when the first marker appeared and terminated when the second marker appeared in feces. All fresh fecal samples were weighed and stored at −20°C immediately after collection.

### Chemical analyses

The collected feces were dried in a forced-air drying oven (FC-PO-150; Dongseo Science) at 55°C until reaching a constant weight and then finely ground less than 1 mm for chemical analyses. The DM (method 930.15), N (method 990.03), ether extract (method 920.39), ash (method 942.05), amylase-treated neutral detergent fiber (method 2002.04), and acid detergent fiber (method 973.18) contents in the commercial diet were analyzed [10]. Fecal samples were also analyzed for DM and N according to the AOAC International [10]. Crude protein (CP) in the commercial diets and fecal samples was calculated by multiplying N content by 6.25. Gross energy of the diet was determined using bomb calorimetry (Parr 6400; Parr Instruments).

### Statistical analyses

Data for daily BW, feed intake, and days of age were collected using the feed intake and BW gain and nutrient requirement models provided by the Korean Feeding Standard for Swine [11]. To determine daily BW and feed intake, sigmoidal models with days of age as the independent variable were developed using the NLIN procedure of SAS (SAS Institute). Based on the total N requirement and DM content in the diet provided by the NRC [1], an equation for estimating the dietary CP content (g/kg DM) was developed using the REG procedure of SAS using BW as the independent variable. The equation for the dietary CP content (g/kg DM) was used for estimating daily N intake which was an independent variable for estimating daily fecal Nex from pigs. The prediction equation for dietary CP content was: dietary CP content (g/kg DM) = 0.0096×BW^2^ (kg)−2.271×BW (kg)+255.12 (r^2^ = 0.978; p< 0.001). The values suggested by the IPCC [8] were compared with those determined in the present study using the TTEST procedure for one-sample t-test in SAS. Statistical significance was declared at p<0.05.

### Models for estimating body weight, feed intake, and fecal excretion

The ATTD of DM and N was determined as described by Kong and Adeola [9]. The sigmoidal model for daily BW of a market pig was developed:


(1) $$\text{BW of a market pig }(\text{kg})=\frac{143.4}{1+{\text{e}}^{-0.029\times (t-124)}}$$

where *t* (day) represents the age of the pig (r^2^ = 0.999; p<0.001). The average slaughter BW of a market pig in Korea was 116 kg [12]. Based on the sigmoidal model for daily BW of a market pig, the age at which pigs reached 116 kg was estimated to be 175 days of age. Daily feed intake (g/day) of a pig was predicted using the following sigmoidal model developed in the present study:


(2) $$\text{Daily feed intake of a market pig }(\text{g}/\text{day})=\frac{2,971}{1+{\text{e}}^{-0.035\times (t-80.5)}}$$

where *t* (day) represents the age of the pig (r^2^ = 0.999; p<0.001). The total feed intake of a pig (kg/pig) was calculated by summing daily feed intake from 0 to 175 days of age. The average daily feed intake of a pig (g/day) was calculated by dividing the total feed intake (kg/pig) by 175. Daily fresh fecal excretion (g/day) during 0 to 175 days of age was estimated using the following equation developed in the present study:


(3) $$\begin{array}{l} \text{Daily fresh fecal excretion }(\text{g}/\text{day})=(100\%-\text{ATTD of DM})\\ \times \text{DM intake}\times \text{Fresh}-\text{to}-\text{dry fecal weight ratio} \end{array}$$

where ATTD of DM (%) and fresh-to-dry fecal weight ratio of pigs were obtained from the present animal experiment and DM intake (g/day) was calculated by multiplying the daily feed intake (g/day) with DM concentration of 0.90. The total fresh fecal excretion (kg/pig) of a market pig was calculated by summing the daily fresh fecal excretion (g/day) from days 0 to 175. The average daily fresh fecal excretion (g/day) was calculated by dividing the total fresh fecal excretion (kg/pig) by 175 days. The representative BW was determined based on the age corresponding to the average daily feed intake (g/day) and fresh fecal excretion (g/day). The representative BW was used for the calculation of annual Nex from pigs.

### Calculations of nitrogen excretion from pigs

The daily fecal Nex (g/day) was estimated by the following calculation:


(4) $$\begin{array}{l} \text{Daily fecal Nex }(\text{g}/\text{day})=\\ \text{Daily N intake}-\text{ATTD of N}\times \text{Daily N intake} \end{array}$$

where daily N intake (g/day) was calculated by multiplying daily feed intake (g/day) and dietary CP content (g/kg DM) divided by 6.25. The ATTD of N (%) was determined from the present animal experiment. Daily urinary Nex was calculated using an equation developed by Vu et al [13]:


(5) $$\begin{array}{l} \text{Daily urinary Nex }(\text{g}/\text{day})=\\ 19.16-0.0627\times \text{Dietary CP content}-0.436\times \text{BW}+\\ 0.00352\times \text{Dietary CP content}\times \text{BW} \end{array}$$

where dietary CP content (g/kg DM) was estimated using the equation developed in the present study using BW (kg) as an independent variable. The total amounts of fecal and urinary Nex (kg/pig) from 0 to 175 days of age were calculated by summing the daily fecal and urinary Nex (g/day) for 175 days. The daily total Nex of a market pig was calculated as:


(6) $$\begin{array}{l} \text{Daily total Nex }(\text{g}/\text{day})=\\ \text{Daily fecal Nex}+\text{Daily urinary Nex} \end{array}$$

where fecal Nex and urinary Nex in g/day.

The annual total Nex from a market pig (kg/year) was calculated by multiplying 365 by the daily total Nex from pigs at the representative BW. The annual total Nex of 14.5 kg/year from breeding pigs, including gestating and lactating sows, was adopted from a previous experiment by a French research group [14] who used diets containing 14.0% CP and 16.5% CP for gestating and lactating sows, respectively. Based on the population ratio of 91:9 for market pigs to breeding pigs in Korea [12], the annual Nex from all pigs was calculated as a weighted mean.

To compare with the daily Nex rate of 0.75 kg/1,000 kg of animal mass suggested by the IPCC [8], the daily Nex rate (kg/1,000 kg of animal mass) for a market pig was calculated using the values obtained in the present work:


(7) $$\begin{array}{l} \text{Daily Nex rate }(\text{kg}/1,000\;\text{kg of animal mass})=\\ \text{Daily total Nex}\times \frac{1}{\text{Representative BW}} \end{array}$$

where daily total Nex in g/day and the representative BW in kg.

## RESULTS

**Figure 1:**
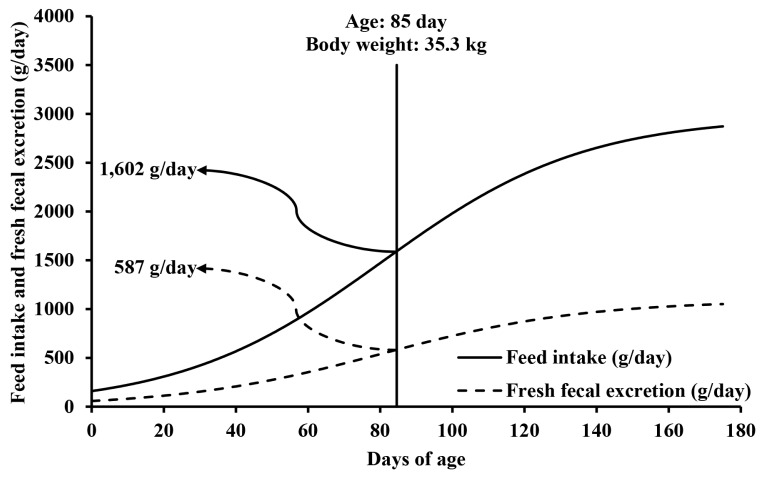
The representative body weight of a market pig based on daily feed intake and fresh fecal excretion. A sigmoidal model for daily feed intake was: Daily feed intake (g/day) = 2,971÷(1+e^−0.035×[^*^t^*^−80.5]^), where *t* represents the day of age (r^2^ = 0.999; p<0.001). Daily fresh fecal excretion of pigs was calculated as: daily fresh fecal excretion (g/day) = (100−Apparent total tract digestibility of dry matter [DM])×DM intake×fresh-to-dry fecal weight ratio, where apparent total tract digestibility of DM and fresh-to-dry fecal weight ratio were 88.4% and 3.55 from the present animal experiment, and DM intake (g/day) was estimated using the NRC model [1]. The total feed intake and fresh fecal excretion during 175 days were 280 and 103 kg/pig, respectively. The average feed intake and fresh fecal excretion were 1,602 and 587 g/day, respectively, corresponding to a pig with body weight of 35.3 kg at 85 days of age.

In the present animal experiment, average ATTD of DM and N for growing pigs fed a commercial diet were 88.4% and 88.8%, respectively (Table 2). The average fresh-to-dry fecal weight ratio was 3.55. During 0 to 175 days of age, the total feed intake, fresh fecal excretion, and Nex of a market pig were estimated to be 280, 103, and 2.9 kg/pig, respectively (Table 3). By dividing the total feed intake and fresh fecal excretion by 175 days, the average daily feed intake and fresh fecal excretion for a market pig were 1,602 and 587 g/day, respectively (Figure 1). Using the average daily feed intake and fresh fecal excretion, the representative BW of a market pig was determined to be 35.3 kg at 85 days of age.

**Figure 2:**
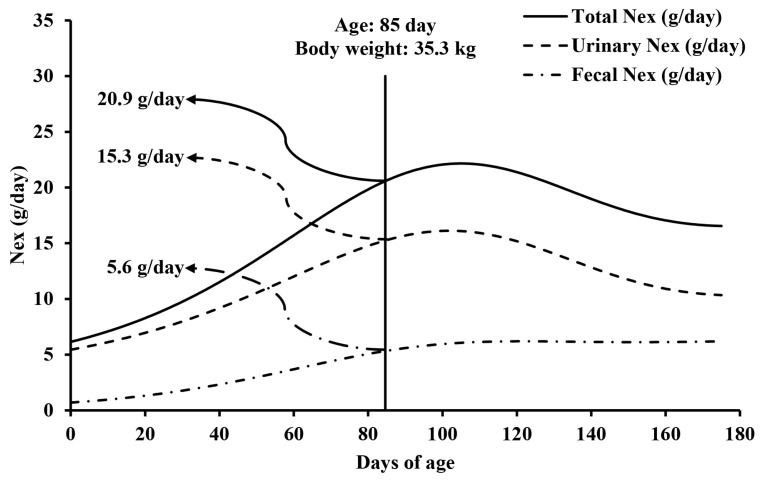
Estimated daily fecal, urinary, and total nitrogen (N) excretion (Nex) of a market pig from 0 to 175 days of age. Daily fecal Nex was estimated as: Daily fecal Nex (g/day) = Daily N intake − Apparent total tract digestibility of N × Daily N intake, where daily N intake (g/day) was calculated using the Korean Feeding Standard for Swine [11] and a prediction equation for dietary crude protein content based on body weight (BW) as an independent variable: dietary crude protein content (g/kg dry matter) = 0.0096×BW^2^ (kg)−2.271×BW (kg)+255.12 (r^2^ = 0.978; p<0.001). Apparent total tract digestibility of N was 88.8% from the present animal experiment. Daily urinary Nex (g/day) was calculated using an equation developed by Vu et al [13]. Daily total Nex (g/day) from a pig was calculated as the sum of the daily fecal and urinary Nex. At a BW of 35.3 kg, which is the representative BWof a market pig, daily fecal, urinary, and total nitrogen excretion from a pig were 5.6, 15.3, and 20.9 g/day, respectively.

The daily fecal, urinary, and total Nex at the representative BW of 35.3 kg were 5.6, 15.3, and 20.9 g/day, respectively (Figure 2). The annual total Nex for a market pig was 7.6 kg/year (Table 3). Considering 14.5 kg/year of annual Nex from gestating and lactating sows, the annual total Nex per pig in Korea was 8.23 kg/year, which was less than the 18.0 kg/year suggested by the IPCC (p<0.001; Table 4). The daily Nex rate for a market pig was 0.584 kg/1,000 kg of animal mass, which was less than that suggested by the IPCC (p<0.001).

## DISCUSSION

Nitrogen in the feces and urine is converted into N_2_O through nitrification and denitrification during pig manure storage and composting [4,7,8]. The N_2_O has the greatest global warming potential among the greenhouse gases produced by pigs, accounting for approximately 51% of total greenhouse gas emissions from swine manure management [6]. The IPCC [8] proposed various methods, including Tier 1, Tier 2, and Tier 3 methods for estimating N_2_O emissions. The Tier 1 method used default values suggested by the IPCC [8], such as the daily Nex rate and representative BW of pigs, to estimate annual N_2_O emissions. In contrast, the Tier 2 and Tier 3 methods used country-specific values to calculate annual N_2_O emissions from pigs rather than using values provided by the IPCC [8]. The Greenhouse Gas Inventory and Research Center of Korea [6] used the Tier 1 method to estimate N_2_O emissions from pigs in the Korean swine industry due to the lack of information on the values specifically for Korea. In 2019, the IPCC [8] used the daily Nex rate and representative BW to provide a more accurate annual Nex from pigs compared with the previous annual Nex estimate of 20 kg/year in the previous edition of the IPCC [7]. However, the annual Nex (18.0 kg/year) from pigs in Western Europe suggested by the most recent edition of the IPCC [8] and potentially applied to Korea has not been validated. In the present study, therefore, the representative BW of market pigs, the daily Nex rate, and the annual Nex suggested by the IPCC [8] were validated and the values for pigs in Korea were determined to improve the accuracy of N_2_O emission from pigs.

In the animal experiment, the diet primarily consisted of corn and soybean meal, which are the major feed ingredients used in the Korean swine industry [11]. The ATTD of DM and N observed in this study was comparable to that of corn-soybean meal-based diets in previous studies for growing pigs [15,16]. The BW of pigs used in the present animal experiment was greater than the estimated representative BW of 35.3 kg. As the intestinal tract of pigs develops with increasing BW [17], the ATTD of nutrients at representative BW of 35.3 kg may differ from that at 56.6 kg BW, which was the initial BW in the animal experiment. However, Xie et al [18] reported that the ATTD of DM and N did not differ across the BW range of 29.0 to 58.6 kg in pigs fed a corn-soybean meal-based diet. Additionally, the study by Zhao et al [19] also showed that the ATTD of DM and N in pigs was not different between BW of 25.0 and 60.0 kg.

The representative BW for a market pig reported by the IPCC [8] was 61 kg, calculated as the average of BW at weaning (7 kg) and at slaughter (115 kg) [20]. However, this BW is not representative as pig BW increases in a quadratic manner as suggested by the NRC [1]. In addition, Nex changes in a non-linear manner as pigs grow [21]. In the present study, therefore, the representative BW was determined as 35.3 kg based on daily feed intake and fresh fecal excretion from birth to slaughter (Figure 1).

The representative BW determined in the present study is well supported by annual swine feed production of 6.7 million ton/year in Korea [12]. In 2024, the population of pigs in Korea was 11.1 million [12] and annual total feed intake of market pigs and sows was calculated as 6.9 million ton/year based on the daily feed intake of 1.6 kg/day at the representative BW of 35.3 kg for 10.1 million market pigs and the daily feed intake of 2.7 kg/day for 1 million gestating and lactating sows. In contrast, if 61 kg BW is used as the representative BW of a market pig [8] with daily feed intake of 2,278 g/day, the annual total feed intake for market pigs and sows would be 9.4 million ton/year which largely exceeds the annual swine feed production in Korea. Thus, the representative pig BW suggested by the IPCC [8] overestimates feed intake, and thus, fecal nutrient excretions. Based on the representative BW of 35.3 kg, the annual Nex of a market pig in Korea was calculated to be 7.6 kg/year which is very comparable to the value of 7.5 kg/year estimated in New Zealand [22]. Instead of using a representative BW, Hill [22] calculated a weighted mean of Nex for 4 growth stages of market pigs.

Based on the sigmoidal model for daily BW and slaughter BW of 116 kg in Korea [12], birth-to-slaughter period of a market pig in the present study was set at 175 days. Approximately two production cycles of a market pig can be carried out per year. As total feed intake from birth to slaughter is 280 kg per market pig (Table 3), annual feed intake of a market pig is calculated to be 560 kg/year. As annual feed intake of 10.1 million market pigs is calculated as 5.7 million ton/year and that of 1 million sows is 1.0 million ton/year, annual total feed intake of pigs is calculated to be 6.7 million ton/year which is identical to the annual feed production in 2024 [12]. Thus, the slaughter BW of 175 days is reasonable based on the feed production statistics in Korea [12].

Although the dietary CP content of 17.0% (as-fed basis) for 35.3 kg BW was based on the NRC [1] in the present study, actual dietary CP concentrations of swine diets in Korea would be less than the NRC [1] values. In 2024, the Ministry of Agriculture, Food and Rural Affairs in Korea established an upper limit of dietary CP in pig diets to address global warming and odor emission issues in pig production [23]. For 35.3 kg BW, the dietary CP should not exceed 15.0% (as-fed basis) which is 2 percentage unit less than the values used in the present study. Kim et al [23] reported that reducing dietary CP from 18.2% to 13.4% (as-fed basis) in growing pigs decreased Nex and odor compounds without adversely affecting growth performance. Similarly, Cho and Kong [24] also reported that reducing dietary CP content from 16.0% to 12.0% (as-fed basis) in growing pig diets did not compromise growth performance. When reducing dietary CP contents, an additional usage of crystalline amino acids is inevitable to meet the standardized ileal digestible amino acid requirements [25]. Consequently, fecal N digestibility will increase due to the high digestibility of crystalline amino acids [26] and urinary Nex will decrease as the quantity of amino acids exceeding the requirements is reduced in pigs fed low-CP diets [27]. Therefore, the actual annual Nex from pigs in Korea may be less than estimated value in the present study due to the regulations on the dietary CP.

Although urinary Nex from market pigs was estimated using the equation developed based on growing-finishing pigs [13], a model for estimating Nex from sows was not available. Thus, annual Nex of 14.5 kg/year from a sow in France suggested by Dourmad et al [14] was adopted in the present study. Further research is warranted on estimating Nex from breeding pigs in Korea.

## CONCLUSION

The representative BW of a market pig was 35.3 kg at 85 days of age based on average feed intake and fresh fecal excretion. Fecal, urinary, and total Nex per pig were estimated at 5.6, 15.3, and 20.9 g/day, respectively. Considering that the Korean swine population consists of 91% market pigs and 9% breeding sows, the weighted mean of the total annual Nex for pigs in Korea was 8.23 kg/year which was less than 18.0 kg/year provided by the IPCC.

## Figures and Tables

**Table 1:** Analyzed energy and chemical composition of a commercial diet (as-fed basis)

Item (%)	Commercial diet^1)^
Gross energy (kcal/kg)	4,038
Dry matter	89.3
Crude protein	17.7
Ether extract	7.0
Ash	5.8
Amylase-treated neutral detergent fiber	15.8
Acid detergent fiber	3.3

All analyses were performed in duplicate.

1) The commercial diet was sourced from Nonghyup Feed.

**Table 2:** Apparent total tract digestibility (ATTD) of nutrients and fecal excretions in growing pigs fed a commercial diet^1)^

Item	n	Mean	SD	Min.	Max.	CV (%)
Initial body weight (kg)	7	56.6	2.3	53.1	59.5	4.0
Final body weight (kg)	7	69.9	1.9	66.9	72.8	2.8
ATTD of dry matter (%)	7	88.4	1.7	86.0	90.7	1.9
ATTD of nitrogen (%)	7	88.8	2.5	85.1	91.6	2.9
Fresh fecal excretion (kg/day)	7	0.80	0.13	0.63	1.01	16.19
Dry fecal excretion (kg/day)^2)^	7	0.22	0.03	0.19	0.28	15.44
Fresh-to-dry fecal weight ratio	7	3.55	0.10	3.38	3.73	2.76

1) The commercial diet was sourced from Nonghyup Feed.

2) The collected feces were dried in a forced-air drying oven (FC-PO-150; Dongseo Science) at 55°C until reaching a constant weight to calculate dry fecal excretion.

SD, standard deviation; CV, coefficient of variation.

**Table 3:** Estimated feed intake, fresh fecal excretion, and total nitrogen excretion (Nex) from day 0 to day 175 and for a year

Item	Feed intake^1)^	Fresh fecal excretion^2)^	Total Nex^3)^
Birth-to-slagughter period (kg/pig)^4)^	280	103	2.9
Annual (kg/year)^5)^	585	214	7.6

1) Feed intake was estimated using the following models: Daily feed intake (g/day) = 2,971÷(1+e^−0.035×[^*^t^*^−80.5]^), where *t* represents the day of age (r^2^ = 0.999; p<0.001).

2) Fresh fecal excretion was calculated as: fresh fecal excretion (g/day) = (100%−Apparent total tract digestibility of dry matter [DM])×DM intake×Fresh-to-dry fecal weight ratio, where apparent total tract digestibility of DM and fresh-to-dry fecal weight ratio were 88.4% and 3.55 from the present animal experiment, and DM intake (g/day) was calculated using the NRC model [1].

3) Total Nex = Fecal Nex+Urinary Nex.

4) The sum of daily feed intake, fresh fecal excretion, and total Nex was calculated over the period from birth to slaughter of a market pig at 175 days of age.

5) The annual daily feed intake and fresh fecal excretion were calculated as: Annual = Sum×365 days÷175 days, and the annual total Nex was calculated by multiplying the daily total Nex of 20.9 g/day at representative body weight by 365.

**Table 4:** Comparison between the present study and the Intergovernmental Panel on Climate Change (IPCC) for representative body weight, daily nitrogen excretion (Nex) rate, and annual Nex values suggested

Item	Present study	IPCC^1)^	p-value
Representative body weight of a market pigs (kg)^2)^	35.3	61.0	-
Daily Nex rate (kg/1,000 kg of animal mass)^3)^	0.584±0.013	0.750	<0.001
Annual Nex (kg/year)^4)^	8.23±0.15	18.03	<0.001

Least squares mean±standard error of the means.

1) The representative body weight, daily Nex rate, and annual Nex provided by the [8] were for Western Europe.

2) Representative body weight was determined based on average daily feed intake of 1,601 g/day and fresh fecal excretion of 587 g/day.

3) Daily Nex rate was calculated as: Daily Nex rate (kg/1,000 kg of animal mass) = Daily total Nex÷Representative body weight.

4) Annual Nex of entire pigs was calculated as the weighted mean for a population consisting of 91% market pigs at 7.6 kg/year and 9% breeding pigs at 14.5 kg/year from Dourmad et al [14].

**Table 5:** Estimated body weight, feed intake, fresh fecal excretion, and total nitrogen excretion (Nex) of a pig at 25 to 175 days of age^1)^

Item	Body weight (kg)	Feed intake (g/day)	Fresh fecal excretion (g/day)^2)^	Total Nex (g/day)^3)^
Age (day)				
25	8.0	361	132	8.9
75	28.3	1,339	490	19.8
125	72.7	2,465	903	23.7
175	116.4	2,872	1,052	20.4

1) Body weight and feed intake were estimated using the following models: Body weight (kg) = 143.4÷(1+e^−0.029×[^*^t^*^ −124]^); Daily feed intake (g/day) = 2,971÷(1+e^−0.035×[^*^t^*^−80.5]^), where *t* represents the day of age (r^2^ = 0.999; p<0.001).

2) Fresh fecal excretion was calculated as: Fresh fecal excretion (g/day) = (100%−Apparent total tract digestibility of dry matter [DM])×DM intake×Fresh-to-dry fecal weight ratio, where apparent total tract digestibility of DM and fresh-to-dry fecal weight ratio were 88.4% and 3.55 from the present animal experiment, and DM intake (g/day) was calculated using the NRC model [1].

3) Total Nex = Fecal Nex+Urinary Nex.

## Data Availability

Upon reasonable request, the datasets of this study can be available from the corresponding author.
